# Depression among Patients with an Implanted Left Ventricular Assist Device: Uncovering Pathophysiological Mechanisms and Implications for Patient Care

**DOI:** 10.3390/ijms241411270

**Published:** 2023-07-10

**Authors:** Hilmi Alnsasra, Fouad Khalil, Radha Kanneganti Perue, Abed N. Azab

**Affiliations:** 1 Cardiology Division, Soroka University Medical Center, Beer-Sheva 8410501, Israel; 2 Faculty of Health Sciences, Ben-Gurion University of the Negev, Beer-Sheva 8410501, Israel; 3 Department of Internal Medicine, University of South Dakota, Sioux Falls, SD 57105, USA; 4 Department of Cardiovascular Medicine, University of Nebraska Medical Center, Omaha, NE 68198, USA; 5 Department of Nursing, Faculty of Health Sciences, Ben-Gurion University of the Negev, Beer-Sheva 8410501, Israel; 6 Department of Clinical Biochemistry and Pharmacology, Faculty of Health Sciences, Ben-Gurion University of the Negev, Beer-Sheva 8410501, Israel

**Keywords:** heart failure, left ventricular assist device, death, depression, quality of life, suicide, treatment

## Abstract

Depression is a common and devastating mental illness associated with increased morbidity and mortality, partially due to elevated rates of suicidal attempts and death. Select patients with end-stage heart failure on a waiting-list for a donor heart undergo left ventricular assist device (LVAD) implantation. The LVAD provides a circulatory flow of oxygenated blood to the body, mimicking heart functionality by operating on a mechanical technique. LVAD improves functional capacity and survivability among patients with end-stage heart failure. However, accumulating data suggests that LVAD recipients suffer from an increased incidence of depression and suicide attempts. There is scarce knowledge regarding the pathological mechanism and appropriate treatment approach for depressed LVAD patients. This article summarizes the current evidence on the association between LVAD implantation and occurrence of depression, suggesting possible pathological mechanisms underlying the device-associated depression and reviewing the current treatment strategies. The summarized data underscores the need for a rigorous pre-(LVAD)-implantation psychiatric evaluation, continued post-implantation mental health assessment, and administration of antidepressant treatment as necessary.

## 1. Depression—Background and Treatment

Depression is a mental illness characterized by a feeling of sadness that is not necessarily related to a direct reasonable cause [1,2]. In addition to a despondent mood, patients may present with a wide variety of symptoms, including a feeling of distress, low self-esteem, changes in appetite, anhedonia, sleep disturbances, low social activity, decreased libido, ideas of self-harm, and suicidal thoughts [1,2]. Depressive disorders adversely affect various aspects of patients’ lives, and generate an enormous burden on caregivers, the health system, and society in general. For example, depression is associated with a prominent negative influence on patients’ quality of life [3]. Moreover, it may lead to cognitive dysfunction [4], which occasionally causes functional and occupational problems [5], over-utilization of health resources, and huge economic costs [6,7].

The etiology and pathophysiological mechanisms underlying depression are not fully understood [8,9]. Several factors have been linked to the pathophysiology of depression, including: psychosocial stressors [10,11], genetic factors [12,13,14], neurotransmitter disturbances [1,2,15,16,17], and inflammatory processes in the brain [18,19,20], among others [1,2,21,22,23,24]. Moreover, precipitating factors, such as chronic somatic illness, emotional stressors, or a family member’s illness or death, may occasionally precede the onset of depression. Importantly, many chronic medical diseases have been suggested as possible causes leading to the development of depression, including cardiovascular disorders [25,26], metabolic ailments (e.g., diabetes mellitus and obesity) [27,28], chronic kidney disease [29], (post-) stroke events [30,31], and other neurological illnesses [32,33], among others. 

Depression is conventionally treated by one, or a combination, of the following three treatments: pharmacotherapy, psychotherapy, and electroconvulsive therapy (ECT) [1,34,35,36]. Different types of psychotherapy can be effective, such as cognitive behavioral therapy or interpersonal therapy [37]. For certain patients, ECT can be a safe and useful treatment option [35,38]. ECT is usually given in severe cases of depression, such as treatment-resistant or psychotic depression, and to patients with suicidal thoughts [35,38]. Nevertheless, although psychotherapy and ECT are administered to a substantial percentage of patients, pharmacotherapy remains the most common therapeutic strategy for the treatment of depression, and includes a wide diversity of medications [2,39,40,41,42]. Nevertheless, despite the evidence attesting to the efficacy of these medications [2,39,41], a large percentage of patients do not respond to treatment, and/or suffer a plethora of unwanted side effects, resulting in low adherence to treatment and relapse of symptoms [2,40,43,44,45,46,47]. These limitations stimulated the search for novel, more effective, and better-tolerated treatments to help a higher percentage of patients. Thus, in recent years, several new therapeutic approaches have been introduced in clinical practice, including the use of the anesthetic drug ketamine [48,49], psychedelic compounds such as psilocybin [50,51], transcranial magnetic stimulation [38,52], and vagal nerve stimulation [53], among others.

## 2. LVAD Implantation in Heart Failure

**Figure 1:**
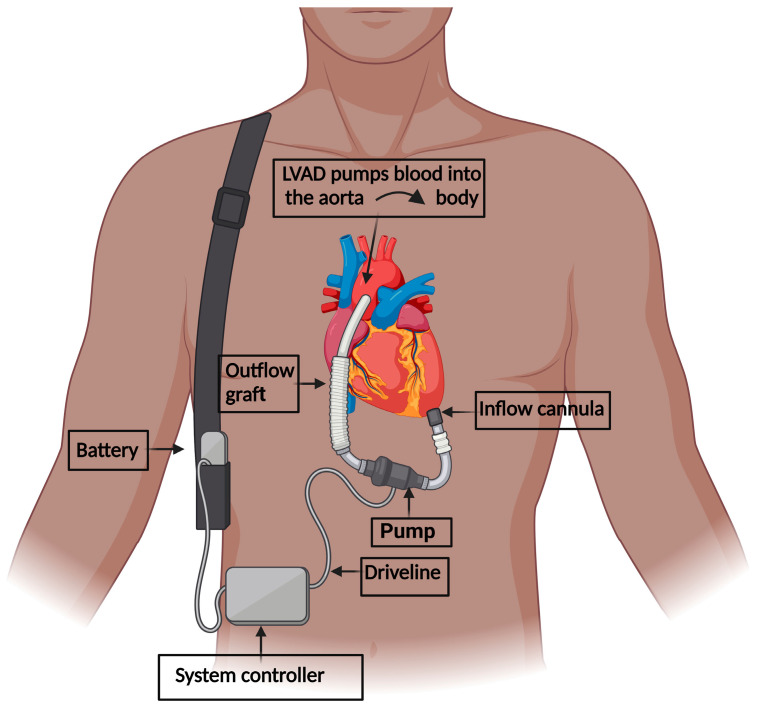
**Elements of the left ventricular assist device.** The inflow cannula is fixed into the left ventricle. The outflow conduit interjoins the ascending aorta. The technology of the pumping chamber is engineered to withdraw blood, which has returned from the lungs and entered the left ventricle, subsequently pumping it through the outflow graft into the ascending aorta, which is then supplied to the body. The driveline rests percutaneously, holding the battery and system controller, supported by a shoulder strap.

Heart failure (HF) is a chronic disease with significant morbidity, disability and a 5-year mortality of about 52.6% [54]. HF affects more than 6.7 million adults in the United States, with the prevalence expected to increase to eight million adults by 2030 [54,55]. While heart transplantation remains the gold-standard therapy for patients with end-stage HF, the total number of donor organs available is limited, and the demand continues to be higher than the supply [56]. The left ventricular assist device (LVAD) is a battery-operated pump implanted inside the heart’s left ventricle, and pumps blood to the rest of the body through the ascending aorta (see Figure 1 for illustration). LVAD has been utilized to keep patients listed for heart transplantation alive until a donor organ is available (bridge-to-transplant therapy), or as a destination therapy in some patients who are ineligible for heart transplants; in this case, patients can receive long-term treatment alongside LVAD for improved morbidity and mortality [57,58].

During the period between 2010 and 2019, INTERMACS reported 25,551 primary isolated LVAD implantations [59]. With improved survival rates among the newer LVADs, there has been a shift in device strategy, with most LVADs now being implanted as a destination therapy. In 2019, 73% of the cases were implanted as a destination therapy, 18% as a bridge to (transplant) decision, and 8.9% as a bridge to transplant [54,59].

## 3. Depression among LVAD-Implanted Patients

### 3.1. Current Evidence of Depression among LVAD-Implanted Patients

With more HF patients receiving LVADs as a destination therapy, and thus living longer, post-LVAD implantation quality of life, including post-implantation psychological well-being, has become an increasingly important consideration. Although depression is well-studied in the general population, less is known regarding the mental health of post-LVAD patients. This is important because depression is associated with worsened morbidity and mortality outcomes in HF patients, including exacerbated HF and increased hospital readmission rates in post-implantation LVAD patients [60]. At least one in five HF patients experiences clinically significant depression, at a rate two times higher than the general population [61]. Furthermore, the prevalence of depression is higher in advanced HF patients, increasing proportionally with a worsening New York Heart Association (NYHA) functional class from about 11% in NYHA class 1 patients to 42% in NYHA class 4 patients [61]. While there is evidence that LVADs improve the quality of life and functional capacity in end-stage HF, robust data is lacking regarding the relationship between LVAD implantation and depression. The available data suggests that depression is common in the LVAD implanted population, both pre-implantation and up to a year post-implantation. However, estimates vary widely [62,63,64,65], and were typically obtained from relatively small samples and by a variety of screening methods. Also, there is a paucity of data regarding the prevalence of depression among long-term follow up post-LVAD implantation. In one observational study with 120 LVAD recipients (median time from implantation 0.82 years), 15% of patients were found to have moderate to severe depression as detected by a self-reported questionnaire [62]. Furthermore, 12% of respondents admitted to having suicidal thoughts [62].

There is conflicting data regarding improvement in depression after LVAD implantation, with some studies showing amelioration of depression within the first year of LVAD implantation and others showing no improvement in depression from baseline (pre-implantation) [66,67,68,69,70]. It is possible that there may also be a temporal association between depression and LVAD implantation, with depression (and anxiety) decreasing over time, at least within the first year after implantation [71]. One small observational study involving 23 patients found that 43% of participants reported antidepressant drugs use pre-LVAD implantation, with a drop in antidepressants usage to 13% of participants at six months post-implantation [71]. While this drop was not statistically significant, the study sample size was small. In a study by Yost et al., in addition to significant reduction in mean Beck Depression Inventory-II (BDI-II) scores, the percentage of patients scoring >14 (suggestive of moderate depression) decreased from 41% pre-LVAD to 18% at follow up 6–12 months post-LVAD implantation (mean follow up 251 days post-implantation) [66]. There is very limited long-term follow-up data regarding depression in LVAD patients. The limited data available have demonstrated that, after initial improvements within the first year, there may be decreased quality of life and increased prevalence of depression beyond one-year post-implantation [67,68]. Possible explanations for this include either hitting a wall, so to speak, in the functional benefits gained with LVAD with patients now feeling better and wanting to do more, but having the limitations of a durable mechanical support, or patients remaining beyond this time point being those who were too sick for transplant or destination therapy [68].

### 3.2. Prognostic Implications

It is well-established that depression is associated with higher rates of mortality and morbidity in HF patients, including worsening HF symptoms, increased hospital re-admission rates, poorer physical and social functioning, and overall lower quality of life [72,73,74,75]. However, less is known about the prognostic implications of depression specifically in LVAD-implanted HF patients.

A psychological assessment is part of the recommended evaluation process for both LVADs and heart transplants. It is hypothesized that depression may negatively impact post-LVAD outcomes, perhaps via compromised device care, hygiene, and compliance with clinic visits and rehabilitation [76]. A study published by Gordon et al. in 2009 found that a history of depression prior to LVAD implantation was found to be an independent risk factor for driveline infection, increasing the risk threefold [76]. However, a more recent study by Köhler et al. [77] found no association between depression and LVAD infections, despite 36% of their subjects having depression or a sub-depressive mood (all assessed by psychiatric consultation). Retrospective analysis of INTERMACS registry data of 2207 patients with psychiatric comorbidities undergoing their first continuous flow LVAD implantation between 2008 and 2017 included 401 patients with severe depression and demonstrated a statistically significant increase in risk of infection, device failure, thrombotic events, rehospitalization, and poor quality of life scores in patients with severe depression [64]. Multiple other studies also support the finding that depression was associated with higher rates of hospital re-admission but not mortality in LVAD-implanted patients [64,78,79].

### 3.3. Suicidal Ideation

An observational study of LVAD-implanted patients showed that, out of 120 patients, 11.6% answered affirmatory to the Patient Health Questionnaire-9 (PHQ-9) item regarding thoughts of suicidal ideation [62]. There are several case reports of LVAD patients attempting to commit suicide by disconnecting their driveline [80]. Analysis of data from the large multicenter ASSIST-ICD (Determination of Risk Factors of Ventricular Arrhythmias After Implantation of Continuous Flow Left Ventricular Assist Device With Continuous Flow Left Ventricular Assist Device) observational study in France found that 10 out of 494 (2.0%) LVAD recipients had attempted or completed suicide over a follow-up period of 18.8 months [81]. Nine of these patients were male (with the caveat that 87% of the sample were male), eight had LVADs implanted as a destination therapy, and only two had a known history of psychiatric disorder, though four did not undergo psychiatric evaluation prior to LVAD implantation. On average, these patients committed suicide 12.5 months post-implantation, and methods included unplugging or severing their LVAD cable or drug intoxication.

## 4. Possible Mechanisms and Pathophysiology of Depression in LVAD-Supported Patients

### 4.1. Psychosocial Factors

**Figure 2:**
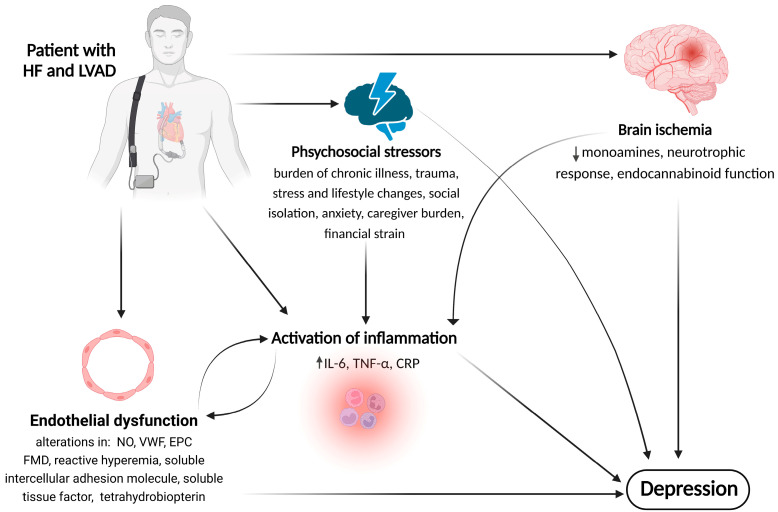
**Illustrative pathophysiological mechanisms of LVAD-induced depression**. The spectrum of potential pathological mechanisms linking LVAD implantation to depression may include patterns relating to psychosocial stressors, endothelial dysfunction, activated inflammatory cascades, and brain ischemia. Importantly, there is interplay between these pathophysiological factors, as they may influence the severity of one another. **↑**—Indicates increase, **↓**—indicates reduction. Abbreviations: CRP, C-reactive protein; EPC, endothelial progenitor cells; FMD, flow-mediated dilation; HF, heart failure; IL, interleukin; LVAD, left ventricular assist device; NO, nitric oxide; TNF, tumor necrosis factor; VWF, von Willebrand factor.

LVAD implantation is a significant life event for HF patients and their loved ones. In addition to continuing to live with a chronic illness after undergoing a major surgery, there is a learning curve in the post-operative period pertaining to device management, including dressing changes and practices to reduce the infection risk, changing the electrical power source, battery management, and troubleshooting alarms (see Figure 2 for illustration). Patients and their care partners may experience stress due to the responsibility and challenges of managing the device and, more fundamentally, also due to the fear and anxiety of depending on an external power source [82,83]. On returning home, living with the LVAD results in permanent changes to daily life and routines, including adjustments to the home environment, and even clothing, to accommodate the new device [84]. Negative body perception, restriction of hobbies such as swimming and contact sports, and unemployment may contribute to social isolation and depression [84,85]. Furthermore, disruptions in sleep and sexual activity are common, and may be associated with increased occurrence/tendency of depression [71,86].

### 4.2. The Role of Inflammation

Despite the beneficial role of LVAD, several studies have illustrated a persistent elevation of inflammatory mediators in patients with HF after LVAD implantation [87]. In addition to the pre-existing systemic inflammatory state due to HF, contact between the blood and the artificial surface might further augment the inflammatory response. Consistently, higher levels of inflammatory mediators, such as the chemokines granulocyte-macrophage colony-stimulating factor, macrophage-derived chemokine, and macrophage inflammatory protein1β, were reported after LVAD implantation [87]. Furthermore, several studies have shown an increase in inflammatory markers, such as interleukin (IL)-6, IL-8, tumor necrosis factor (TNF)-α, monocyte chemoattractant protein-1, and C-reactive protein (CRP), due to upregulation of the renin–aldosterone angiotensin system secondary to the non-physiologic flow pattern [88,89,90,91]. Considering the mounting evidence suggesting that inflammation plays a significant role in the pathophysiology of depression [18,19,20,92,93,94], it is reasonable to assume that inflammation also contributes to the pathophysiology of depression in LVAD-implanted patients (Figure 2).

Increased inflammatory markers, including CRP, IL-6, TNF-α, and IL-1β, have been reported in patients with depression [18,19,20,92,93,94]. Moreover, high levels of inflammatory mediators in the blood have been associated with a lower response to antidepressant treatments [95]. Consistent with the “depression-inducing” effects of pro-inflammatory cytokines, particularly TNF-α, several lines of evidence attested to the efficacy of selective TNF-α antagonists as a potential treatment for depression [96,97]. It has been hypothesized that cytokines alter the synthesis and reuptake of dopamine, norepinephrine, and serotonin in the brain via different pathways [98,99]. Moreover, increased levels of pro-inflammatory mediators, such as IL-1β, IL-6, and TNF-α, have been linked to diminished neurogenesis and abnormal behavioral phenotypes in rats [100]. Furthermore, a recent study demonstrated a strong association between inflammation and aggressive behavior [101]. Subjects with aggressive/explosive behavior were found to have elevated plasma levels of inflammatory markers such as CRP, IL-8, and TNF-α. This is particularly important, as aggressive/explosive behavior increases the risk of suicidal ideation and attempts [102,103,104] and, thus, may be a predictor for suicidal behavior among LVAD-implanted patients. 

Dantzer et al., proposed a neural pathway where the locally produced cytokines can stimulate primary afferent nerves, such as the vagus nerve, and eventually alter brain chemistry and lead to depression via immune-to-brain communication [105]. Another proposed mechanism is the humoral pathway, where Toll-like receptors on macrophages produce inflammatory cytokines that cross the blood–brain barrier (BBB) by diffusion [106]. A third pathway includes transportation of cytokines across the BBB via cytokine transporters [107]. Notably, TNF-α reduces tight junction protein expression and increases the permeability of the BBB. This facilitates the entry of both cytokines and immune cells into the brain [108].

Immune-to-brain communication through these pathways leads to inflammatory cytokine production in the brain by microglial cells and, ultimately, the development of depressive symptoms [105]. TNF-α also activates microglial cells with a subsequent release of glutamate [109], and higher levels of glutamate lead to neuronal injury through excitotoxicity [110]. Furthermore, IL-1β was found to decrease neurogenesis in hippocampal progenitor cells through activation of the kynurenine pathway [111]. Kynurenine is metabolized in a regulated fashion to either quinolinic acid, which is excitotoxic via agonistic effect at N-methyl-D-aspartate (NMDA)-glutamate receptors, or to kynurenic acid, which is neuroprotective and acts as an antagonist on NMDA receptors [112]. With inflammation, there is a loss of this balance and predominance of the excitotoxic pathway, with a subsequent loss of brain volume reported in depressed patients [113]. In general, inflammation disrupts BBB and induces functional and structural changes in the central nervous system [114]. These changes are thought to induce neuronal damage, decrease hippocampus neurogenesis, and impact long-term synaptic potentiation and brain global connectivity, all of which cultivate the development of depressive behavior. 

### 4.3. The Role of Endothelial Dysfunction (ED)

Vascular endothelium has a vital role in cardiovascular homeostasis through the regulation of cell adhesion, platelet aggregation, angiogenesis, vascular tone, permeability, and fibrinolysis. The reduced pulsatility in the contemporary LVADs with the associated increased shear stress result in endothelial dysfunction and decreased production of endothelial-derived vasodilatory substances, such as nitric oxide (NO) [115,116]. Furthermore, elevated inflammatory mediators in the setting of HF and LVAD might contribute to the progression of ED in these patients [87,117]. Endothelial function can be measured by different techniques based on different physiological concepts, such as vascular tone, post-occlusive reactive hyperemia, and dynamic permeability [118]. Flow-mediated dilation (FMD) is a commonly used technique to measure arterial dilation during post-occlusive reactive hyperemia. Although the exact pathophysiology or causal relationship between ED and depression remains contestable, there is accumulating evidence linking ED to depression [119,120]. Chrysohoou et al. [121] comprehensively examined the literature investigating the mediating role of ED and depression, proposing that the evident mechanisms plausibly include elements pertaining to circulating endothelial progenitor cells, FMD, amplified sympathetic arousal, a persistent noradrenaline response to stress, and abundant circulating catecholamines. To specify, studies have demonstrated an inverse relationship between FMD and depression, suggesting an association between depression and cardiovascular disease mediated by ED [122]. Using FMD as a measure, a meta-analysis demonstrated a 1.4% lower dilating response in depressed patients compared to controls [120]. Other markers of ED, such as low reactive hyperemia, endothelial-derived NO, von Willebrand factor, soluble intercellular adhesion molecule, soluble tissue factor, and tetrahydrobiopterin, have been associated with depression [121,123]. Furthermore, there is evidence supporting the improvement in endothelial function with antidepressants [124]. Nevertheless, future studies are needed to confirm and characterize the relationship between ED and depression.

### 4.4. The Role of Brain Ischemia

The incidence of stroke in LVAD patients ranges from 0.08 to 0.29 events per patient-year, with some variation depending on the type of implanted device [125,126,127,128,129]. Cerebrovascular pathology, in the form of hemorrhage and infarcts, is almost universal in LVAD patients, as evidenced by autopsy studies [130,131]. The impaired cerebral autoregulation is thought to be a major contributor to the development of cerebrovascular events in LVAD patients [132,133]. Impaired autoregulation of cerebral blood flow results in total dependence on blood pressure [134]. In such circumstances, low blood pressure can result in hypoperfusion, while hypertension can cause cerebral hyperemia; these changes can predispose brain damage [135]. The non-physiological flow with the subsequent von Willebrand factor deficiency in LVAD-supported patients might contribute to increased stroke risk in these patients. Although the pathophysiology of stroke in LVAD patients is not well delineated, risk factors include prior strokes, atrial fibrillation, diabetes mellitus, hypertension, and tobacco use [129,136,137]. Operative techniques during the time of implantation can impact the risk of stroke [138,139]. Pump thrombosis, infection, and antithrombotic medications can contribute to the stroke risk in the post-implantation phase.

A high percentage of stroke survivors develop clinical depression, with estimates ranging between 20 to 75% of patients [31,140,141], especially among females [141]. Although the pathophysiology of post-stroke depression is not completely understood, some contributing brain changes have been described. Acute ischemic injury to the monoaminergic neurons in the brain reduces monoamines levels in the frontal cortex, leading to a depressed mood [142]. Low monoamine levels in the reward system and in basal ganglia are associated with anhedonia, fatigue, impaired cognition, and mood changes [142,143]. Another mechanism is altered growth/survival of neurons (neurotrophic response). The neurotrophic response normally increases acutely following ischemic injury to neurons as a compensatory process [142,144]. A normal neurotrophic response with increased neurotrophins, such as brain-derived neurotrophic factor, especially in the hippocampus and prefrontal cortex, is protective against post-stroke depression [145]. Similarly, the decreased neurotrophic response is associated with increased post-stroke stress and poor response to antidepressants [143,146]. Additionally, stroke is associated with an acute inflammatory response that might contribute to the development of depression, as discussed above. Finally, an increasing body of data suggests that altered endocannabinoid system in the brain and mitochondria metabolism might contribute to post-stroke depression [147].

Although these proposed mechanisms (psychological stressors, inflammation, ED, cerebral ischemia) were discussed separately, it should be noted that they have very complex pathways with significant overlaps (Figure 2). Moreover, in some circumstances, such as LVAD driveline infections, all three mechanisms might be activated simultaneously. In this regard, it is well known that infection is associated with systemic inflammation. At the same time, LVAD infection is associated with increased stroke incidence, partially via hypercoagulability and platelet activation [148,149]. Finally, infection also is associated with ED, with subsequent atherosclerosis, vasculitis, and coagulopathy.

## 5. Conclusions 

There is a paucity of knowledge regarding the treatment of depression among LVAD-implanted patients. Currently, it is not known whether a particular treatment strategy is advantageous over other available remedies. Most of the limited existing data are based on observational studies and case reports [63,86,150,151,152,153,154]. The current knowledge suggests that the management of depressed-LVAD-implanted patients does not differ greatly from that given to “regular” patients with depression; namely, that most patients are treated with antidepressant medications, while psychotherapy and ECT are rarely used [86,150]. Nonetheless, considering the unique postulated pathophysiological determinants that contribute to the development of post-LVAD implantation depression (see Section 4), it is possible that the treatment approach in these patients should also be different and take into account their inimitable socio-medical condition. For example, in consequence of the recognized increased inflammatory load in these patients—which seemingly contributes to the development of depression among them—it may be of therapeutic value to consider adding an anti-inflammatory medication to the treatment regimen. However, such a therapeutic intervention would necessitate evidence-based assurance because, under certain circumstances, activation of the immune system and an accompanying low-magnitude inflammatory response may actually benefit the brain [155,156]. In this regard, it has been postulated that the mechanism underlying the therapeutic outcomes of ECT (and other electrical-stimulating interventions, such as deep brain stimulation) involves induction of a neuroinflammatory response, which facilitates homoeostatic and neuroprotective processes in the brain [157,158]. Importantly, the management of such patients should comprise a multidisciplinary approach and involve a cardiologist, psychiatrist, nurse-specialist, psychologist, and social worker, among others as necessary. The evaluation of such patients must consider several important factors, such as: the general medical condition of the patient; onset, duration and severity of depressive symptoms; history of psychiatric illness and others measures of mental status and quality of life; special pre-and-post-implantation events (such as a loss of a family member or significant change in marital status or social condition); past and/or current antidepressant or other psychotropic treatment; and presence of suicidal thoughts and/or suicidal attempts. Thus, in addition to a rigorous assessment of the patient’s condition and the administration of antidepressants, the treatment may necessitate the use of other approaches, including psychotherapy and ECT, or even admission to a psychiatric facility [153] in cases of severe depression or imminent risk of suicide. Clearly, the lack of evidence regarding established therapeutic intervention(s) for the treatment of depression in LVAD-implanted patients underscores the need for more research, and the necessity of conducting prospective randomized clinical trials for elucidating the most effective and safest treatment for this unique patient population.

## Data Availability

Not applicable.
